# Gene functioning and storage within a folded genome

**DOI:** 10.1186/s11658-017-0050-4

**Published:** 2017-08-29

**Authors:** Sergey V. Razin, Sergey V. Ulianov

**Affiliations:** 1 Institute of Gene Biology, Russian Academy of Sciences, Vavilov Street 34/5, 119334 Moscow, Russia; 2 Lomonosov Moscow State University, Biological Faculty, Leninskie Gory 1, building 12, 119192 Moscow, Russia

**Keywords:** Active chromatin, TADs, Hi-C, Self-organization, Epigenetic regulatory mechanisms, Enhancers

## Abstract

In mammals, genomic DNA that is roughly 2 m long is folded to fit the size of the cell nucleus that has a diameter of about 10 μm. The folding of genomic DNA is mediated via assembly of DNA-protein complex, chromatin. In addition to the reduction of genomic DNA linear dimensions, the assembly of chromatin allows to discriminate and to mark active (transcribed) and repressed (non-transcribed) genes. Consequently, epigenetic regulation of gene expression occurs at the level of DNA packaging in chromatin. Taking into account the increasing attention of scientific community toward epigenetic systems of gene regulation, it is very important to understand how DNA folding in chromatin is related to gene activity. For many years the hierarchical model of DNA folding was the most popular. It was assumed that nucleosome fiber (10-nm fiber) is folded into 30-nm fiber and further on into chromatin loops attached to a nuclear/chromosome scaffold. Recent studies have demonstrated that there is much less regularity in chromatin folding within the cell nucleus. The very existence of 30-nm chromatin fibers in living cells was questioned. On the other hand, it was found that chromosomes are partitioned into self-interacting spatial domains that restrict the area of enhancers action. Thus, TADs can be considered as structural-functional domains of the chromosomes. Here we discuss the modern view of DNA packaging within the cell nucleus in relation to the regulation of gene expression. Special attention is paid to the possible mechanisms of the chromatin fiber self-assembly into TADs. We discuss the model postulating that partitioning of the chromosome into TADs is determined by the distribution of active and inactive chromatin segments along the chromosome.

This article was specially invited by the editors and represents work by leading researchers.

## Background 

The concept of hierarchic chromatin organization in the eukaryotic cell nucleus has been developed rather long ago and is commonly accepted now [1–3]. A matter of debate is what the levels are in the hierarchic packaging of the chromatin fibril. It is beyond doubt that the first level of DNA packaging in chromatin is DNA wrapping around a histone octamer to produce a nucleosome. The so-called 10-nm chromatin fiber thereby forms, having a characteristic beads-on-a-string structure. The 10-nm fiber was believed for a long time to coil somehow into a more compact 30-nm fiber. The process is readily detectable in experiments in vitro. Several models were proposed to explain the structure of the 30-nm chromatin fiber, and the best known of them are a one-start solenoid with six nucleosomes per helix turn [4] and a two-start helix with a zigzag nucleosome arrangement [5, 6]. Recent studies showed clearly that even in vitro the 30-nm chromatin fiber is a dynamic structure with a number of conformations converting into one another [7, 8]. Electrostatic interactions between nucleosomes play a key role in the formation of a 30-nm fibril, positively charged N-terminal histone domains (histone tails) of one nucleosome interacting with a negatively charged acidic patch on the surface of another nucleosome [9–12]. It is important to note that histone acetylation substantially reduces the positive charges of the N-terminal tails of histones H3 and H4 and thereby weakens the electrostatic interactions that stabilize the 30-nm chromatin fibril [13]. In a domain model of genome organization [14, 15], histone acetylation-dependent transitions between more and less compact modes of chromatin fibril folding are thought to provide a mechanism that activates or inactivates chromatin domains [16].

It was always clear that higher-order compaction levels must follow the 30-nm chromatin fiber, but the mode of chromatin packing at these levels was long unknown. One of the most common model suggestes that 30-nm fibers are organized in loops, which are attached to the nuclear matrix [1, 17–20]. The question as to whether the loops correspond to functional genome domains was intensely discussed in the literature (for a review, see [21]).

## Current views on the hierarchic levels of chromatin compaction

Several studies published in the recent years questioned the existence of 30-nm chromatin fibrils in living cells [22–26]. A principal problem in studying the higher-order levels of chromatin compaction by electron microscopy is that images of individual chromatin fibers superimpose on one another and thus hinder a configuration analysis of individual fibers. The problem was solved using electron spectroscopy, which makes it possible to examine electron spectroscopic images [27], and electron tomography techniques [28, 29]. Regular 30-nm fibers were not observed in cell nuclei with these new methods. Chromatin mass consisted of tightly associated nucleosome strings (10-nm fibers). The nucleosome packing density differed between euchromatic and heterochromatic regions, but no regular supernucleosomal structure was detected [26, 30]. Similar conclusions were made in an earlier chromatin structure analysis by cryoelectron microscopy [24].

**Fig. 1:**
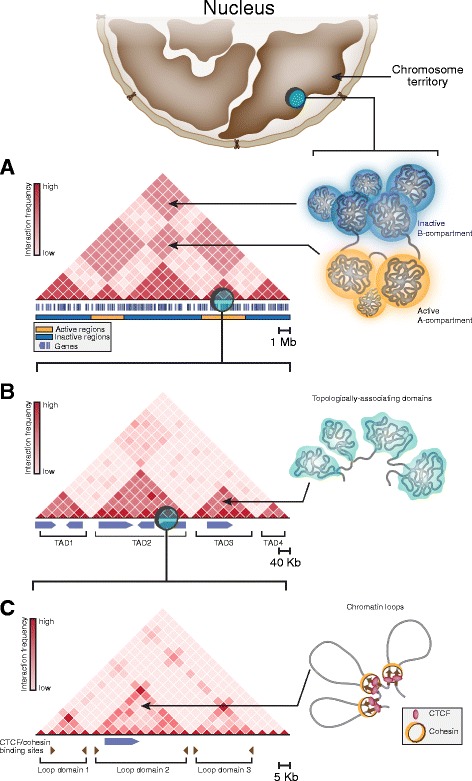
A scheme illustrating the hierarchical structure of interphase chromatin. Chromosome territories (at the top of the picture) are partitioned into A- and B-compartments (**a**) formed by long-range spatial interactions between distant genome loci and containing active and repressed genome regions, respectively. At a sub-megabase level, chromatin is folded into topologically-associating domains, TADs (**b**), commonly interpreted as self-interacting globular structures those positions are largely conserved across cell types. The internal structure of TADs is represented by arrays of so-called loop domains formed by spatial contacts between CTCF/cohesin-binding sites (**c**). Color intensity on illustrative Hi-C maps (on the left side of each panel) reflects average interaction frequency between corresponding genomic bins

The above results do not contradict the mere existence of higher-order hierarchic levels in chromatin compaction, but indicate that these levels are not based on assembly of regular structures, such as the 30-nm fiber. An important contribution to understanding the principles of hierarchic chromatin folding was made in studies that employed the so-called C methods, which address the physical proximity of particular genome regions in the three-dimensional space of the cell nucleus. The methods are based on ligation of DNA fragments located close together. The procedure was proposed as early as the 1990s [31, 32], but did not find broad application until a chromosome conformation capture technique was developed [33]. A Hi-C method assesses the physical proximity of various DNA fragments on a genome-wide scale and is the most informative for analyzing the general principles of chromatin folding [34]. Studies with this experimental technique provided independent experimental support to the existence of chromosome territories [34], which were earlier detected by confocal microscopy of nuclei stained with sets of chromosome-specific hybridization probes [35–37]. In addition, mammalian chromatin was demonstrated to include two compartments, active A and inactive B, which correspond to euchromatin and heterochromatin in the first approximation [34] (Fig. 1a). Finally, chromosome partitioning into the so-called topologically associating domains (TADs) was observed (Fig. 1b). A main feature of TADs is that intra-TAD spatial contacts between genome elements are significantly more frequent than inter-TAD contacts [38–40]. Early studies already showed that profiles of chromosome partitioning into TADs are quite conserved among cells of different lineages and, within syntenic regions, among closely related species [38, 41, 42]. However, the degree of this conservatism is limited. In mammals, 60-80% of the TAD boundaries coincide in cells of different lineages [38, 43]. In *Drosophila*, the number of TAD boundaries coinciding in embryonic and culture cells constitutes 40-50% [40, 44]. Substantial differences in TAD profiles may arise, for example, from differential activation of tissue-specific genes in cells of different lineages [44]. It should also be noted that TADs themselves are organized hierarchically and may have several levels of smaller contact domains separated by weaker boundaries [43–45]. As the resolution of Hi-C maps improved (up to kilobase and even sub-kilobase scale (bioRxiv 149,344; bioRxiv 115,063) that seems to be a natural limit for Hi-C resolution dictated by the average size of restriction fragments generated by a 4-cutter), contact subdomains were observed within mammalian TADs, and many of them were identified as chromatin loops with bases containing CTCF sites and highly enriched in cohesin [46] (Fig. 1c). It should be noted that the level at which contact domains should be termed TADs is still unclear [47]. TADs are most commonly thought to range from 1 million to several millions of base pairs in mammalian cells [38, 39, 41], while the average TAD size is several hundred thousands of base pairs in *Drosophila* [40, 48]. Contact domains with a certain similarity to mammalian and *Drosophila* TADs were observed in plants [49] and lower eukaryotes [50]. However, a number of parameters (stability, size, and genome coverage) substantially differ between these contact domains and TADs present in mammalian and *Drosophila* chromosomes.

What TADs are as physical bodies is an open question, although they are usually equated with chromatin globules detectable using various microscopic techniques [51–53]. This interpretation is partially supported by the results of in situ hybridization with probes distributed through the length of an individual TAD [54].

## TADs are structural and functional domains of the genome

The question of whether structural organization of the genome coincides with its functional organization has been debated in the literature over many years (for a review, see [55]). The problem is difficult to solve because both functional and structural domains of the genome still lack a clear definition. At least two types of functional domains can be identified, namely, those associated with replication and transcription. As for replication, a replicon seems reasonable to consider a functional domain. However, while alternative origins of replication exist and replicon positions are unstable over cell generations [56, 57], other replication domains attract attention. Replication time zones are sufficiently stable in each particular cell type [58]. A good correlation between TADs and extended replication time zones was demonstrated in several studies [59–61].

**Fig. 2:**
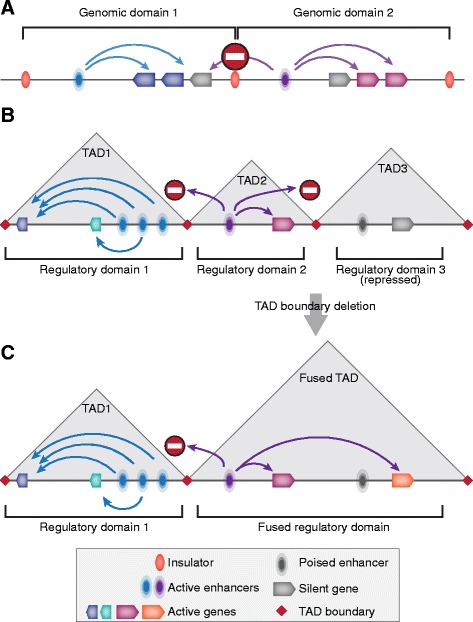
Chromosome partitioning into TADs reflects genome partitioning into regulatory domains delimiting zones of enhancer influence. Conventional concept of genomic domain implies that the entire genome is partitioned into non-overlapping parts (domains) containing gene clusters and regulatory regions (**a**), and demarcated with insulators preventing cross-talk between regulatory systems of the adjacent domains. According to current views, zones of enhancer influence (regulatory domains) largely overlap with TADs (**b**) that spatially confine communication between genes and enhancers located within adjacent regulatory domains. Deletion of TAD boundary leads to TAD fusion and, consequently, to fusion of corresponding regulatory domains resulting in abnormal enhancer-promoter communication and transcription dysregulation (**c**)

In the case of transcription, the definition of a functional domain is also not a trivial question. Before the era of whole-genome research, a limited number of genomic models were used in the majority of experimental studies, the mammalian and avian globin gene loci being the most common ones [16, 62–64]. A gene cluster with distant regulatory elements that control its genes was usually understood as a genome domain in those studies (Fig. 2a). In some cases, this functional domain colocalizes with a chromatin domain demarcated by insulators and exhibiting differential DNase I sensitivity, which correlates with the transcription status of the gene cluster [62, 65]. It is clear now that this definition of a functional domain is simplified. Genome-wide studies showed that one enhancer may activate many genes that do not form a single cluster and are far away from the enhancer along the DNA molecule (Fig. 2b). Enhancers were at the same time found to be far more numerous than known genes, indicating that several enhancers may apparently control the function of one gene [66, 67]. Although the mechanism of action is unclear for enhancers, the most common model postulates that an enhancer should be in direct contact with a promoter to ensure its activation and that the intervening segment of the chromosome fiber loops out to bring the two elements close together [68]. If so, the network of functional relationships between enhancers and promoters must be reflected in a network of physical contacts between respective regions of the chromatin fiber. Networks of contacts between distant genomic elements were detected in fact [67, 69]. They lay at the basis of the so-called regulatory domains (regulatory archipelagos), wherein the majority of genes display a similar expression pattern, which depends on the type of cell differentiation [70, 71]. The above TAD properties clearly indicate that the potential for enhancer–promoter communication is restricted to a TAD because relatively rare contacts arise between genomic elements that belong to different TADs. Colocalization was, in fact, demonstrated for regulatory domains and TADs [71] (Fig. 2b). When TADs fuse as a result of a deletion of the spacer between them, the sphere of influence changes for relevant enhancers (Fig. 2c), leading in some cases to various disorders due alterations in gene expression regulation within the TADs involved [72–74].

Additional line of evidence supporting the idea that TADs represent structural and functional units of the genome arises from the studies of cell differentiation and reprogramming. In the model system of ESC differentiation into several distinct lineages, TADs were found to be largely stable along the genome, but demonstrated a high flexibility in both inter- and intra-TAD interactions [75]. TADs containing upregulated genes exhibit a substantial increase in chromatin interactions and relocate into A-compartment, whereas TADs harboring downregulated genes tend to decrease a number of chromatin contacts and undergo A-to-B compartment switching.

It should be noted that the establishment of enhancer–promoter communication should depend on how fast the enhancer and its target promoter are brought close together in the nuclear space. A restriction of the search area to a TAD will certainly reduce the time it takes to establish enhancer–promoter communication. Lack of rigidity in the TAD structure is of importance in this context. Alternative configurations of the chromatin fiber continuously interchange within a TAD [76]. This is likely to provide additional possibilities for cell adaptation to changing environment [77].

## Mechanisms underlying the formation of topologically associated domains

Many models were proposed in the literature to describe the mechanisms of TAD formation. Computational simulations showed that entropic forces primarily drive the formation of compact contact domains in a polymer model confined to a limited space. The profile of polymer partitioning into contact domains may further be modulated by additional factors, such as bridges between distant polymer regions [78]. The finding that the physical properties of a polymer confined to a limited space play a key role in the formation of contact domains agree well with the fact that contact domains occur in one or another form in the genomes of various organisms, including bacteria [79], and special cell types, such as spermatozoa, which contain protamines in place of histones in their nuclei [80].

**Fig. 3:**
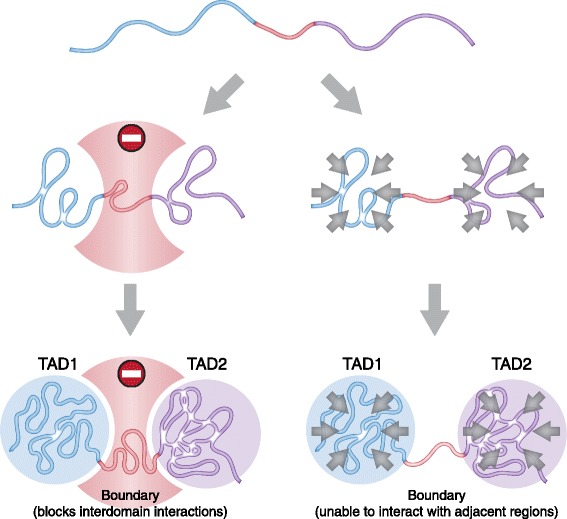
A scheme illustrating two proposal mechanisms of TAD boundary action. *Left panel*: boundary plays an active role in TAD demarcation preventing interdomain interactions. *Right panel*: boundary represents a genomic region unable to fold into higher-order structures and/or to interact with adjacent regions. In contrast, TAD is comprised of chromatin regions which tend to interact with each other forming globular structures

It is crucial to understand what factors determine the relatively specific profiles of chromosome partitioning into TADs. Two alternatives are possible here (Fig. 3). One is that boundary elements exist to prevent the spatial interactions between the chromatin fiber segments separated by the elements. The other alternative suggests that there are chromatin fiber segments that are capable of folding into compact (e.g., globular) structures annotated as TADs on Hi-C maps and chromatin fiber segments that cannot fold into such structures because of their certain physical specifics.

It is assumed in the boundary element hypothesis that insulators play a main role in TAD separation [81–83]. Insulators were discovered 25 years ago as genomic elements that block the interaction between an enhancer and a promoter when located between them and prevent the spread of inactive chromatin domains [84, 85]. As became clear recently, the functions of insulators are diverse and are based on their capability of closing a chromatin fibril in loops [86–88]. Insulators accordingly came to be considered as architectural elements of the genome, and insulator-biding proteins are often termed the architectural proteins [89, 90]. Several such proteins are found in *Drosophila* [91, 92]. In mammals, CTCF in the only known insulator protein [93, 94]. CTCF maintains the spatial organization of the genome by acting alone or recruiting cohesin [95–98].

The CTCF ability to organize DNA in loops certainly contributes to the TAD formation. High-resolution Hi-C maps constructed for various human and mouse cells [46] were collated with CTCF genomic positions, and CTCF was implicated in the formation of the majority of the so-called loop domains. However, holding compact chromatin masses together rather than partitioning them is the case here. Both loop and ordinary domains are present in TADs [46]. The latter lack loop structures. A deletion of an extended chromatin fiber fragment that occurs at the boundary between two TADs and harbors a CTCF binding site was reported to cause partial TAD fusion [39]. However, the deletion could involve not only the CTCF binding site, but also other genomic elements important for TAD separation. CTCF depletion was not observed to cause a dramatic reorganization of TADs [99]. On the other hand, it seems likely that DNA-associated CTCF preserves its association even when the CTCF concentration in the cell is substantially reduced via RNA interference. Experiments with a controllable CTCF degradation system showed that a substantial decrease in CTCF, including the CTCF bound to DNA, leads to a considerable TAD loosening (a decrease in TAD insulation) [100]. It should be noted that mammalian cells were used in virtually all experiments that demonstrated an important role of CTCF in determining inter-TAD positions. The role that CTCF plays in *Drosophila* is less clear. In particular, loop domains limiting spatial contacts between distant genomic elements were not observed in *Drosophila* cells. We studied the distributions of several insulator proteins relative to TAD boundaries in four *Drosophila* cell lines of different origins and did not detect an appreciable enrichment in binding sites for dCTCF and Su(Hw) for TAD boundaries [44]. On the other hand, data from our and other studies indicate that TAD boundary regions harbor transcribed genes and are enriched in histone modifications typical for active chromatin [40, 44, 48]. TADs usually contain tissue-specific genes, which are not transcribed in the majority of cell types. Comparisons of the profiles of chromosome partitioning into TADs in various cell lines showed that transcriptional activation of tissue-specific genes correlates with a loosening of the respective TAD or its separation into two TADs with an intensely transcribed gene between them [44]. The observations made it possible to assume that inactive segments of a chromatin fiber spontaneously fold into TADs. A compact TAD arrangement is due to electrostatic interactions between nucleosomes belonging to different fibers [44]. Entropic forces induced by macromolecular crowding may further stabilize the association of chromatin fibers in TADs [101]. The potential to form various conglomerates is well known for nucleosome fibers. The conglomerates are stabilized by interactions between positively charged N-terminal tails of histones H3 and H4 and a negatively charged patch on the surface of a nucleosomal globule [10, 12]. The same interactions facilitate the formation of 30-nm nucleosome fibers at low fiber concentrations, when inter-fiber contacts are unlikely [11, 102]. As was already mentioned above, histone acetylation, which is typical for active chromatin, decreases the histone charge and prevents internucleosome interactions [13, 103]. Any active chromatin region of a sufficient length will therefore insulate TADs, the extent of insulation depending on the region length and the extent of histone acetylation. Thus, the distribution of active and inactive genes along a DNA molecule may determine the profile of chromosome organization in TADs. We checked this assumption by computer simulation of self-folding of a virtual polymer that consists of alternating nucleosome blocks of two types reproducing the properties of active and inactive chromatin regions [44]. Nucleosomes of “inactive” blocks were capable to establish relatively unstable contacts with nucleosomes of the same type. Nucleosomes of “active” blocks, which were shorter in size, were incapable of establishing contacts with each other and nucleosomes from inactive blocks. Model polymeric chains organized in this manner were observed to form globular structures, which consisted of nucleosomes from inactive blocks [44]. It is essential to note that inactive nucleosomes could establish contacts with both nucleosomes of the same inactive block and nucleosomes of other inactive blocks in our model. As a result, conglomerates of inactive nucleosomes fused to produce super-conglomerates in some cases. In some other cases, nucleosomes of one inactive block formed more than one conglomerate with less compact spacers between the conglomerates. The results of 12 modeling experiments generally differed in the detail of final spatial structures. However, when the data were averaged over all experiments, the resulting Hi-C map contained contact domains (TADs) that coincided with inactive nucleosome blocks and were separated by spacers of active nucleosomes. The spatial genome organization in single cells has been reported to date in two publications. Their results indicate that, indeed, the chromosome partitioning profile obtained experimentally for a cell population is a superposition of many individual configurations, which may substantially differ from the average profile [104, 105]. Our model of TAD organization has an apparent advantage of being based on the well-known properties of nucleosomes and nucleosome fibers. Saturated interactions assumed for nucleosomes are an essential feature of our modeling; i.e., the number of contacts possible for a nucleosome is limited (to one contact in the simplest case). Saturation is quite rapidly achieved in these conditions and is due to contacts between closely spaced nucleosomes. Contacts with distant nucleosomes (including those from different blocks) are not prohibited, but are far rarer for purely stochastic reasons. The so-called volume interactions, which are in no way determined by the known properties of nucleosomes, were assumed in many earlier models of nucleosome fiber behavior [106]. With volume interactions included in the model, the fiber coils into a single globule as soon as equilibrium is achieved [107]. In the above-discussed model, we did not take into account the architectural proteins as we did not found strong enrichment of Drosophila TAD boundaries with deposition sites of CTCF or other known architectural proteins [44]. This observation is in good agreement with the fact that *Drosophila* does not have loop domains [108] that are easy to see on high-resolution Hi-C maps of the human genome [46].

Other models of TAD formation emphasize the role of architectural proteins, which are thought to pull parts of a linear segment of a chromatin fiber together to produce a compact TAD by interacting with each other. To explain the existence of isolated TADs, the models assume a multiplicity of architectural protein groups, each ensuring the formation of a particular TAD [109–111]. The models seem implausible biologically because architectural proteins are 100 times fewer than TADs even in *Drosophila*, which is known to have several architectural proteins in addition to CTCF.

If TADs indeed are predominantly inactive chromatin domains separated by active regions, then the TAD size must depend in a certain way on the gene sizes, the gene distribution through the genome, and the relative sizes of the active and inactive genome fractions. Indirect evidence for this assumption can be found in the literature. For instance, the average size of contact domains is 2–10 Kb in *Saccharomyces cerevisiae* [50], in which a major part of the genome is active and genes are relatively small. Classical TADs were similarly not observed in *Arabidopsis thaliana* [49, 112], whose genome is comparable in size with the *Drosophila* genome, while annotated genes are almost twice as many as in *Drosophila*.

## Specifics of mammalian TADs

As mentioned above, genome organization in contact domains is hierarchic. The question of the level at which contact domains should be considered to be TADs or sub-TADs is solved to a great extent intuitively, based on the common views of average TAD sizes in various organisms [113]. In mammals, the average TAD size is thought to be in the range of one to several thousand Kb [90]. TADs of this size may include many (up to several tens in some cases) sub-TADs [46, 66, 75]. Sub-TADs are often bounded by CTCF binding sites and correspond to the loop domains identified using high-resolution Hi-C map of the human and mouse genomes [46] (Fig. 1c), whereas TAD boundaries are enriched not only with CTCF binding sites, but with tRNA genes, SINE retrotransposons, housekeeping genes and active histone marks H3K4me1 and H3K36me3 as well [38]. Interestingly, the last three properties of TAD boundaries are not mammal-specific. In *Drosophila,* promoter-specific H3K4 monomethylation, ubiquitously transcribed genes and P-element integration events are highly enriched within TAD boundaries [44], denoting the presence of basic features of TAD boundaries such as high transcription level and open chromatin state. Sub-TADs can substantially vary in transcription intensity and chromatin type. The orientation of the CTCF binding sites located at the bases of loops is important for loop formation. Chromatin loops form most often between convergent CTCF binding sites and are lost when the orientation of the sites is changed by gene-engineering manipulations [114]. A model of TAD and sub-TAD formation by loop extrusion assumes that chromatin fiber looping is driven by certain molecular machines, such as a cohesin-involving complex [115, 116]. The capability of looping DNA was demonstrated for cohesins and condensins experimentally [117]. The machines are thought to stop functioning at occupied CTCF binding sites. Another mechanism of an active looping of chromatin fibers is based on the function of RNA polymerase immobilized in transcription factory [118]. The site of RNA polymerase loading on DNA is rendered fixed by CTCF and cohesin, while transcribed DNA is looped out until RNA polymerase encounters the next CTCF binding site. The mechanism agrees well with the existence of genome-wide low-level transcription [119], clusters of similarly oriented genes, and an asymmetric distribution of transcription starts in DNA loops bounded by CTCF sites [120].

We think that genetic information that is not in demand is stored in TADs in the simplest variant. This TAD function is prevalent in *Drosophila* [44]. However, genome partitioning into relatively isolated structural domains came to be useful for organizing the function of regulatory mechanisms as the genome size dramatically increased in mammals and several other vertebrates. As regulatory networks grow in complexity and many distant enhancers arose, TADs acquired another important function of compartmentalizing regulatory elements of the genome to restrict their spheres of influence to particular groups of genes. Several other advantages can be assumed for the partitioning of a large genome into relatively isolated domains. For instance, the time it takes to establish enhancer–promoter communication is substantially shorter. Mechanisms that would move an enhancer to its target promoter in a directional manner are currently not known to exist. Enhancers and promoters move stochastically within the nucleus, and their movements are limited by overall chromatin motility [121]. A genome locus is capable of scanning over 0.5–0.8 μm per hour according to current estimates [121], and this rate is sufficient for an enhancer and a promoter to meet within one TAD. If a whole chromosome territory is to be scanned at the same rate, an enhancer and a promoter will hardly meet within the duration of one cell cycle. Compact chromatin organization in TADs should limit the movements of broken DNA ends when a break occurs within a TAD, thus facilitating their correct ligation via nonhomologous end joining. On the other hand, the inter-TAD location renders active genes more accessible to various damaging agents, and broken DNA ends in inter-TADs should have a far greater mobility than within a TAD. This circumstance should facilitate repair errors, potentially producing fusion genes.

## Concluding remarks

Although the importance of the 3D genome organization for regulation of gene expression has long been envisaged [122], the experimental analysis of this organization became possible only when the appropriate tools were developed, such as 3C and derivative genome-wide procedures [123]. Recent studies clearly demonstrated the functional significance of the spatial contacts between remote genomic elements [124–126]. In addition, our understanding of the levels of DNA packaging has undergone significant changes. With the emergence of 3D genomics it became possible to revisit some long-standing models, such as the domain model of eukaryotic genome organization [55]. Analysis of interaction frequencies of the remote genomic elements allowed to identify self-interacting chromatin domains,TADs [39] which appear to represent structural-functional domains of eukaryotic genome [71, 127]. Mechanisms of TADs assembly remain largely unclear. It is likely, that various factors contribute to their assembly. Our current results strongly suggest that, in *Drosophila*, inactive chromatin domains became assembled in compact masses (TADs) due to electrostatic interaction of nucleosomes located on neighboring fibers [44]. These domains are separated by segments of chromatin fiber that harbor active genes. These segments remain relatively extended because highly acetylated nucleosomes of active chromatin lost the ability to interact with each other. The whole process of TADs formation appear to be stochastic and TAD profiles seen on Hi-C maps emerge only as a population average. In mammals, TADs are much larger and appear to be more complex [43, 113]. While, in Drosophila, the primary function of TADs appears to be the storage of inactive genes [44], mammalian TADs acquire additional function in transcriptional control [118]. Although stochastic interactions of neighboring nucleosomes are likely to contribute also in the assembly of mammalian TADs, the insulator protein CTCF plays an essential role in the spatial and functional separation of these TADs. It has been suggested that chromatin loop extrusion plays an essential role in the formation of mammalian TADs [115, 116]. However, the nature of extrusion machines remains elusive and the model still lacks direct experimental proves. Mammalian TADs have a complex structure and are likely to be assembled from smaller looped and ordinary domains [46]. The relation of these nested domains to the functional organization of the genome remains to be studied.

## Abbreviations

3C: Chromosome conformation capture
ESC: Embryonic stem cells
Hi-C: High-throughput chromosome conformation capture assay
Kb: Kilobases, thousands of base pairs
TAD: Topologically associating domain
